# Knowledge Summaries for Comprehensive Breast Cancer Control: Feedback From Target Audiences in Kenya

**DOI:** 10.1200/JGO.18.00119

**Published:** 2019-01-28

**Authors:** Mishka Kohli Cira, Jo Anne Zujewski, Allison Dvaladze, Nathan R. Brand, Amanda L. Vogel

**Affiliations:** ^1^ Clinical Monitoring Research Program Directorate, Frederick National Laboratory for Cancer Research sponsored by the National Cancer Institute, Frederick, MD; ^2^ Leidos Biomedical Research, National Cancer Institute Campus at Frederick, Frederick, MD; ^3^ University of Washington, Seattle, WA

## Abstract

**PURPOSE:**

Comprehensive breast cancer control programs are needed to decrease breast cancer mortality, but few tools exist to assist stakeholders in limited-resource settings. The Knowledge Summaries for Comprehensive Breast Cancer Control (KSBCs) are a series of evidence-based publications intended to support cancer control planning at various resource levels. The goals of this evaluation research study were to learn about the extent to which the KSBCs could be useful to policymakers, health care providers, and breast cancer advocates in Kenya, and whether introducing the KSBCs led to their uptake, and if so, how they were used.

**METHODS:**

This study used one-on-one interviews, focus groups, and self-administered online surveys. Policymakers were recruited from the Ministry of Health. Providers were recruited from four hospitals in two cities, Nairobi and Eldoret, and one rural municipality, Kijabe. Advocates were recruited from cancer advocacy organizations.

**RESULTS:**

Twenty individuals participated in the research. They found the KSBCs to be educational reference tools that create a shared planning-related knowledge base among diverse stakeholders. The KSBCs were seen to be applicable to a variety of contexts and stakeholders.

**CONCLUSION:**

This study found that the KSBCs can be useful as both an educational tool and a convening tool for multistakeholder engagement in breast cancer prevention and control in a variety of settings. Additional engagement with users of the KSBCs can provide more knowledge about how the KSBCs are used and how they contribute to building collaborations across stakeholder groups to strengthen breast cancer prevention and control in low-resource settings.

## INTRODUCTION

Breast cancer is the most common cancer in women worldwide, with an estimated 1.6 million patients globally in 2012 and a projected 2.2 million patients globally in 2025.^1^ Case fatality rates for breast cancer generally are highest in low- and middle-income countries as a result of a confluence of factors, including poor awareness of breast cancer signs and symptoms, late-stage diagnosis, and multiple constraints at the health system and patient level resulting in limited access to treatment.^2^ Addressing these challenges requires a multipronged approach to breast cancer prevention and control that considers population awareness, early diagnosis, treatment, and palliative care. Collaborations across a variety of stakeholder groups, including policymakers, health care providers (HCPs), and advocates, can be effective in supporting such an approach, because each group contributes its own perspectives and expertise.

Informational and planning materials are needed that provide a shared knowledge base about the evidence-based best practices and planning considerations for these diverse stakeholder groups. Few resources are available to guide a range of stakeholder groups in advocating for comprehensive breast cancer prevention and control, as evidenced by the 2015 review by the American Cancer Society of cancer-related information, education, and communication (IEC) materials in Kenya.^3^ The review identified 18 breast cancer–related IEC materials (15% of total IEC materials), but none of them addressed breast cancer policy and program development for a variety of stakeholder audiences.

The Knowledge Summaries for Comprehensive Breast Cancer Control (KSBCs) were developed to provide members of these varied stakeholder groups with the knowledge and evidence they need to participate as vested partners in breast cancer prevention and control, specifically in low-resource settings.^4^

Knowledge summaries, which serve to summarize evidence-based practices to inform policy and program decision making, have been developed for use by a wide range of stakeholders to address a variety of public health issues. Examples include the World Health Organization Tobacco Knowledge Summaries^5^ and the Partnership for Maternal, Newborn and Child Health Knowledge Summaries.^6^

The KSBCs are a series of 14 publications that summarize the evidence base on seven major topics in breast cancer control: planning, prevention, early detection, diagnosis, treatment, palliative care, and survivorship (Table 1). They were developed by experts from the Union for International Cancer Control, Breast Health Global Initiative, Pan American Health Organization, and Center for Global Health of the US National Cancer Institute (CGH/NCI). A detailed description of the development, content, and pilot testing of the KSBCs has been provided in a prior publication.^4^

**TABLE 1:**
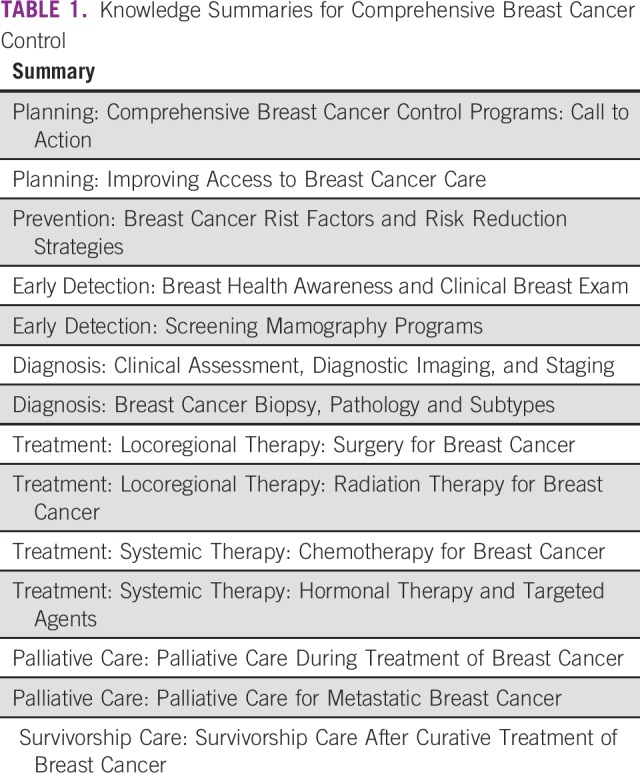
Knowledge Summaries for Comprehensive Breast Cancer Control

The KSBCs recommend adoption of breast cancer prevention and control measures through a phased implementation approach, guided by available resources.^7^ For example, standardized guidelines and capacity to provide diagnostic and treatment services should be in place before considering the introduction of a breast cancer screening program.^7^ The KSBCs focus on providing recommendations for countries with limited resources and tailor recommendations to four levels of resource availability—basic, limited, enhanced, and maximal—reflecting levels described in resource-stratified guidelines developed by the Breast Health Global Initiative.^8^ Each KSBC highlights the need to plan systematically, guiding the user through the following steps, outlined in sections entitled Points for Policymakers:

1. Preplanning
2. Planning step 1: Where are we now? (investigate and assess)
3. Planning step 2: Where do we want to be? (set objectives and priorities)
4. Planning step 3: How do we get there? (implement and evaluate).

In 2017, shortly after the launch of the KSBCs, CGH/NCI conducted a research study to learn more about the potential usefulness of the KSBCs as a resource for collaboration across stakeholder groups to advocate for country-level enhancements in breast cancer prevention and control in Kenya.

This study expanded on prior pilot testing of the KSBCs, specifically by including representatives of all of the intended target audiences for the KSBCs: breast cancer advocates, policymakers, and HCPs. Findings are reported here, followed by a discussion of implications for use of the KSBCs as a policy advocacy tool.

## METHODS

This research study had two specific aims:

1. to learn the extent to which members of the KSBC target audiences in Kenya felt the KSBCs could be useful to their cancer prevention and control–related activities; and
2. to learn whether introducing the KSBCs to these individuals led to uptake, and if so, how these individuals used the KSBCs.

The study design consisted of in-person one-on-one interviews and focus groups with members of the key target audiences for the KSBCs, policymakers, HCPs, and advocates, followed by 3- and 6-month follow-up surveys of interview and focus group participants. The interviews and focus groups addressed specific aim 1, whereas the surveys addressed specific aim 2.

### Setting

Recent policy developments in Kenya reflect a national commitment to cancer control. The Kenyan Ministry of Health and National Cancer Institute of Kenya, through a multistakeholder participatory process, recently revised the National Cancer Control Strategy for 2017 to 2022.^9^ In addition, the Kenyan National Hospital Insurance Fund, which now covers some breast and other cancer treatment modalities, has been tasked with engaging with cancer stakeholders to ensure adequate planning for increased service availability per the National Cancer Control Strategy.^9^

The need for enhanced breast cancer prevention and control in Kenya is evident. Breast cancer is the most common cancer among Kenyan women, with an estimated 4,465 new patients each year.^1^ A study about lay perception of breast cancer in Western Kenya concluded that an overall lack of knowledge and several misconceptions about breast cancer exist.^10^ In addition, there is a high rate of late-stage diagnosis, as reported in 2010 by Otieno et al^11,12^ that more than 70% of women seeking breast cancer care in the Kenyan public sector had a delay of greater than 3 months from symptom onset to diagnosis.

The lack of policy advocacy materials specific to breast cancer prevention and control in Kenya, combined with the recent visible national commitments to cancer control and related stakeholder engagement, created an opportunity to introduce the KSBCs to a multistakeholder audience of policymakers, HCPs, and breast cancer advocates in Kenya.

### Sampling Strategy and Recruitment

CGH/NCI used a convenience sample to recruit participants from the target audiences for the KSBCs (policymakers, HCPs, and advocates) through established CGH/NCI contact networks in Kenya. CGH/NCI has collaborated with the Kenyan Ministry of Health, National Cancer Institute of Kenya, and stakeholders to promote increased coordination of Kenyan cancer research and control activities since 2013.^13^ Policymakers were recruited through direct contact from the research team. HCPs were recruited by direct contact or through referral from CGH/NCI contacts at hospitals that varied in their level of available resources to diagnose and treat cancer. Three hospitals were located in Nairobi (population of more than 6.6 million), one hospital was located in Eldoret (population of 300,000), and one hospital was located in Kijabe (population of less than 20,000). Breast cancer advocates were recruited through contacts at the Kenyan Network of Cancer Organizations, a Nairobi-based membership organization of community-based cancer organizations in Kenya. All participants received an invitation letter and written disclosure statement describing the study goals, methods, and voluntary nature of their participation.

### Data Collection

Policymakers and HCPs took part in one-on-one in-person 30- to 60-minute interviews. Interviews were conducted at their places of work. To prepare for their interviews, policymakers and HCPs were asked to review two of the KSBCs, including *Planning: Improving Access to Care* and one KSBC of their choosing.

Breast cancer advocates participated in a 35- to 45-minute in-person semistructured focus group or a 30-minute one-on-one in-person semistructured interview. Interviews and focus groups were conducted at the Kenyan Network of Cancer Organizations offices. To prepare, breast cancer advocates were asked to review two KSBCs, *Planning: Improving Access to Care* and *Early Detection: Breast Health Awareness and Clinical Breast Exam.*

The interview and focus group guides included a core set of questions asked of all participants and additional questions specific to the target audience (policymaker, HCP, or advocate). The core questions addressed usefulness, strengths, and limitations of the KSBCs to inform breast cancer control planning activities, and relevance of the KSBCs to the Kenyan context and to the target audiences.

All participants agreed to have their interviews or focus groups audio recorded and agreed to receive a follow-up self-administered online survey at 3 and 6 months after the initial interaction. The self-administered online survey included questions about whether and how the participants had used and/or shared the KSBCs since the initial interaction. The US National Institutes of Health Office of Human Subjects Research Protections approved this study (OHSRP Determination 17-NCI-00043-1).

### Data Analysis

Interviews and focus groups were audio recorded and professionally transcribed. Two of the authors (M.K.C. and J.A.Z.) used thematic coding to analyze the transcripts.^14^ Codes were developed using a combined inductive and deductive approach.^15^ The self-administered online survey data were analyzed manually by M.K.C.

## RESULTS

Twenty individuals participated in the study via 10 one-on-one interviews and two five-person focus groups. The interview participants included seven individuals whose primary work was as HCPs, two^2^ individuals whose primary work was as policymakers, and one^1^ breast cancer advocate. All 10 focus group participants were active in breast cancer advocacy. Twelve participants (60%) responded to the 3-month follow-up survey, and four participants (20%) responded to the 6-month follow-up survey. Two main themes were identified from the interviews, focus groups, and follow-up surveys. These main themes, and the subthemes within them, are described below.

### The KSBCs Are Educational Reference Tools That Create a Shared Planning-Related Knowledge Base Among Diverse Stakeholders

#### The KSBCs as a learning tool.

The participating advocates reported that the KSBCs have a rich level of detail that is useful to build policy advocacy knowledge and to better understand the full continuum of cancer prevention and control. One advocate stated that the KSBCs help advocates to better apply their knowledge for policy advocacy:

What the Knowledge Summaries have done is graduated [me] from that basic understanding of cancer, to a better understanding of how [to] frame that into a policy question.

At least two of the HCPs stated that it is useful as a clinician to read about nonclinical considerations, such as a patient’s finances and other barriers to care. One HCP stated:

So there’re all these barriers and one of the things I learned was…how do you try and make it possible for a woman—how do you attract a woman to come to care, and thinking through about finances and health insurance? And how can you sort of integrate that?

Both policymakers stated that reading about the diagnostic recommendations was helpful for bridging the gap between what the clinicians know and what the policymakers need to know to build effective programs.

Of the 12 study participants who responded to the 3-month follow-up survey, seven (58%) used or referenced the KSBCs since their first introduction to the KSBCs, and eight (66.6%) shared the KSBCs with at least one other person. The top reason cited for continued use of the KSBCs was to obtain information for advocacy efforts (n = 6). One HCP reported that the KSBCs were shared with junior colleagues “as a starting point for breast cancer management,” and one reported that they were incorporated into the pathology resident training curriculum.

#### The KSBCs as a planning tool.

Every interview and focus group included remarks on the usefulness of the KSBCs as a planning tool. Six of seven participating HCPs and both participating policymakers commented on the relevance of both the resource-stratified approach and the phased implementation approach to decision making in the Kenyan context. One HCP noted that the phased implementation approach in the KSBCs helps with thinking in terms of feasibility:

[it] tells you what works and what doesn’t work. And I think, as a planner or administrator, if you were to read this, then you know what not to do and what to do.

One policymaker stated that the KSBCs were useful at a health systems level in terms of creating an understanding of the “linking [of] the various components of a breast cancer program.” Both participating policymakers noted that the Points for Policymakers boxes helped them to better understand how to decide where to begin in terms of integrating breast cancer into implementation of the National Cancer Control Strategy.

### The KSBCs Are Applicable to a Variety of Contexts and Stakeholders

#### Applicability to the Kenyan context.

Participating advocates, one of the two policymakers, and six of the seven HCPs commented on the applicability of the KSBCs’ content in Kenya. One advocate stated that the KSBCs, which are written for use in a variety of low-resource settings, would lose their broad appeal if tailored to one country or cultural setting. One policymaker stated that it is up to local stakeholders to use the KSBCs as a guide for their programming purposes. This was repeated by three of the HCPs.

One HCP stated that even within a country (such as Kenya), there are cultural variations, and it is important for the user to adapt the information accordingly. In a similar vein, one policymaker pointed out that the KSBCs put emphasis on understanding what is culturally appropriate to a given setting when developing a program or intervention:

you could have very good ideas but you go to specific parts of the country and, if it’s not culturally appropriate, it’s not going to work.

#### Applicability to a wide range of stakeholders.

Breast cancer advocates and one policymaker stated that the KSBCs are written in a way that is comprehensible and well suited to their needs. One HCP stated that the KSBCs lay out information that is relevant to an array of audiences:

everyone can pick what is of their interest and then, obviously, there are online resources and other stuff that you can go in[to] and take it further.

In addition to being relevant for advocates, policymakers, and HCPs, participants suggested that the KSBCs would be helpful to patients, patient navigators, caregivers, survivors, and community leaders.

## DISCUSSION

An increasing number of resources and tools exist to guide specific target audiences in decision making for cancer control. Policymakers look to World Health Organization frameworks and toolkits.^16^ HCPs refer to clinical guidelines (eg, NCCN Guidelines and Clinical Resources^17^). Advocates refer to globally available advocacy materials (eg, *Supporting National Cancer Control Planning: A Toolkit for Civil Society Organizations [CSOs]*).^18^ The KSBCs add value as an evidence-based, adaptable, decision-making resource for a wide range of stakeholders working specifically in breast cancer prevention and control in low-resource settings.^4,19^

The results of this study illustrate that the KSBCs provide information that is useful to policymakers, HCPs, and advocates alike and therefore the KSBCs can be a useful tool for facilitating multistakeholder dialogue for planning purposes. Even though this was most participants’ first exposure to the KSBCs, they uniformly understood that this was a tool for cancer prevention and control planning for a variety of stakeholder groups. The example of the HCP who discussed addressing a patient’s nonclinical needs indicates an opportunity for advocates to work with HCPs to ensure that a broader range of patients’ needs, relevant to their cancer care, are addressed. Likewise, the policymakers stated that exposure to the clinical recommendations in the KSBCs improved their ability to engage with clinicians to address relevant technical components of breast cancer prevention and control. In addition, follow-up survey respondents reported that they shared the KSBCs with others and used them for their own work, which indicates the value of the KSBCs.

One of the overarching recommendations throughout the KSBCs is that users of this planning resource can use the KSBCs to guide them in identifying locally relevant data and information for decision making. This recommendation was understood by the participants in this study, despite this being their first exposure to the KSBCs. Participants from each of the target audiences noted that it would be limiting if a resource was developed solely for use in one country or setting and that the KSBCs’ guidance for how to access locally relevant data were valuable to them.

The primary limitation of this study was the small sample size. Although there were notable individual comments about the KSBCs, the sample size limited the ability to make generalizable observations or recommendations. A second limitation was the convenience sampling method, which may have biased the findings by working with individuals known through contacts or directly by the research team. Another potential limitation was the fact that the participants were responding to questions about the KSBCs immediately after their initial introduction to the resource. The use of the follow-up survey somewhat addressed that limitation, but the follow-up survey itself had a low response rate. Despite these limitations, study participants shared rich observations about the value and utility of the KSBCs, and their reports of subsequent dissemination and use of the KSBCs underscore these observations.

Given that the KSBCs were developed for use in a variety of low-resource settings and that the Kenyan target audiences participating in this study found them to be relevant and useful, findings from this study may be relevant to similar settings in other low- and middle-income countries.^20^ Future engagement with users of the KSBCs can provide more knowledge about how the KSBCs are used by a range of stakeholders and how they contribute to building collaborations across stakeholder groups to strengthen breast cancer prevention and control in low-resource settings.

The KSBCs are informational policy and planning tools designed to support efforts to strengthen comprehensive breast cancer control through a phased implementation, resource-appropriate approach. Findings from this study demonstrate that the KSBCs, which are designed for use in a variety of settings, are seen as useful by policymakers, HCPs, and breast cancer advocates in Kenya, a middle-income country in sub-Saharan Africa that is in the process of strengthening its national cancer control program. Additional engagement with users of the KSBCs will provide further insight into the KSBCs as tools to support multisectoral engagement in cancer control planning in diverse settings.
